# Structural, Compositional, and Dielectric State Profiling in Label‐Free Single‐Cell Monitoring

**DOI:** 10.1002/smtd.70759

**Published:** 2026-06-03

**Authors:** Changi Baek, Youngho Song, Seongcheol Park, Sujin Hyung, Sang Eun Yoon, Yong Jae Shin, Soo‐Yeon Cho

**Affiliations:** ^1^ School of Chemical Engineering Sungkyunkwan University Suwon Republic of Korea; ^2^ Division of Hematology‐Oncology Department of Medicine Samsung Medical Center Sungkyunkwan University School of Medicine Seoul Republic of Korea; ^3^ Samsung Precision Genome Medicine Institute Samsung Medical Center Seoul Republic of Korea

**Keywords:** imaging cytometry, impedance cytometry, label‐free single‐cell monitoring, vibrational spectroscopy

## Abstract

Individual cells sense and transition between functional states, and the distribution of these states over time determines drug response, disease progression, and cell manufacturing outcomes. However, repeated measurement is difficult with label‐based acquisition, as photobleaching, phototoxicity, and probe‐induced perturbation accumulate. Label‐free monitoring that leverages intrinsic physical signals circumvents these constraints, shifting the analytical burden from label chemistry to instrumental drift. In this review, we organize this field into three measurement modalities, including imaging‐based, vibrational spectroscopy‐based, and electrical sensing‐based, each linked to distinct intrinsic state variables. Anchoring each modality to biological state variables such as structural organization, molecular composition, or dielectric architecture enables a physics‐grounded framework that clarifies how intrinsic signals map onto functional cellular phenotypes in longitudinal monitoring. We describe the measurement principle, drift sources, and feature space for each modality, and evaluate representative platforms against a frame spanning design, feature definition, quantitative performance, and validation practice. Dominant analytical constraints differ systematically across modalities, motivating integrative architectures in which complementary modalities resolve ambiguities that no single modality can disentangle. We further discuss shared requirements for calibration, standardized reporting, and multimodal integration, and outline requirements in hardware miniaturization, edge inference, and artificial intelligence‐guided molecular attribution to support scalable quantitative single‐cell phenotyping.

## Graphical abstract

Label‐free single‐cell monitoring leverages distinct physical interactions to access structural, compositional, and dielectric states of cells, enabling non‐perturbative, repeatable, and information‐rich measurements across diverse biological contexts. This review organizes representative platforms by intrinsic state variables and connects measurement physics to practical descriptors, drift sources, throughput limits, and specificity, reframing the field and outlining an outlook for integrative and longitudinal monitoring.

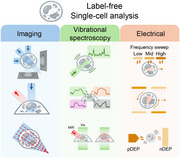

## 1 Introduction

Living organisms derive their functional properties from the collective behavior of individual cells. Because each cell independently senses, adapts, and transitions between states, the distribution of cellular states and its temporal evolution are central to the organism‐level phenotype. However, most experimental measurements do not resolve individual cells and instead report population‐averaged signals. Bulk measurements average across millions of cells and compress this underlying distribution, concealing the structured phenotypic heterogeneity that governs functional outcomes [1, 2]. Bulk averaging produces two mechanistically distinct classes of analytical failure. First, rare subpopulations that disproportionately drive biological outcomes, such as drug‐resistant clones or tumor‐initiating cells, contribute little to the population mean and can remain undetectable by bulk readouts despite their functional dominance [3]. When these subpopulations go unrecognized, pre‐existing resistant cells can expand under selection pressure and drive treatment failure or disease recurrence [4, 5]. Second, the population average itself may correspond to no individual cell in the distribution, so bulk measurements can report a state that no cell actually occupies, misrepresenting the biological landscape in the opposite direction [6]. Single‐cell monitoring addresses this gap by tracking how individual cellular states evolve over time and in response to defined perturbations such as drug treatment or environmental stress. Because these distributions evolve, questions including recovery kinetics, phenotypic drift, and subpopulation emergence require measurement systems that can be applied repeatedly without converting technical variation into apparent biological change [7]. To support such longitudinal inference, monitoring requires repeated measurements whose extracted features remain quantitatively comparable across time, cell conditions, and batches within a consistent coordinate system, a requirement substantially more stringent than single‐timepoint profiling [8, 9].

Conventional approaches to single‐cell analysis have addressed this need primarily through label‐based flow cytometry, in which cells are pretreated with fluorescent dyes or antibody conjugates that bind specific molecular targets, and the intensity of the resulting optical signal is used to infer protein expression and molecular state [10]. Fluorescent labels and affinity probes provide molecular specificity, yet their repeated use introduces compounding measurement failures. Photobleaching arises when repeated optical excitation drives irreversible photochemical reactions that disrupt the fluorophore's conjugated electronic structure, shifting its absorption spectrum and quenching emission [11]. As a result, the same cell may appear to change state because the label signal collapses rather than because the underlying biology has changed [12]. Phototoxicity also arises because excitation generates reactive oxygen species and local heating that perturb membranes, proteins, and DNA, thereby altering the very state trajectory being measured and introducing systematic bias into longitudinal readouts [13]. These degradation mechanisms limit temporal sampling frequency and reduce the number of time points that can be acquired from a single cell without compromising biological validity [14]. Furthermore, label‐based single‐cell monitoring tools are inherently constrained by the number of spectrally distinguishable fluorophores that can be used simultaneously, limiting the dimensionality of the measured state space [15]. Affinity probe delivery also requires membrane permeabilization or incubation steps that disrupt the physiological state of the cell, precluding longitudinal tracking of the same cell across multiple time points under native conditions.

Label‐free monitoring leaves cells unmodified by affinity tags or engineered genetic modifications, instead reading physical signals that cells emit or modulate intrinsically. The absence of exogenous chemistry simplifies sample preparation, shortens test time, and eliminates label‐induced signal degradation under repeated acquisition, shifting the analytical challenge from controlling label chemistry to controlling instrumental drift and ensuring that extracted physical features remain stable across sessions [16, 17, 18]. In many implementations, cells remain viable after measurement, enabling downstream culture, sorting, or functional assays that connect physical trajectories to biological outcomes. This reduced perturbation is particularly valuable for fragile or activation‐prone cells, where added reagents can alter membrane state or metabolic activity and obscure intrinsic dynamics. Because label‐free signals originate from the cell's own physical properties, this approach provides direct access to cell structural, compositional, and dielectric features that reflect the physiological state of individual cells [19]. Cell structural features such as size, shape, volume, and refractive index (RI) report spatial organization and dry mass distribution [20]. In parallel, cell compositional features such as lipid, protein, and nucleic acid content report the molecular inventory, while cell dielectric features such as membrane capacitance and cytoplasmic conductivity report the electrochemical architecture of cellular compartments [21]. These features are quantitative indicators of cell cycle progression, metabolic activity, membrane integrity, and phenotypic identity.

These three state variables are accessed through three physically distinct measurement families, each grounded in a different physical interaction with the cell [22]. Cell structural state variables, including RI and morphology, capture spatial organization and are accessed primarily through imaging‐based platforms such as quantitative phase imaging (QPI) and holotomography, which interrogate cells with coherent light and recover structural information from the resulting phase or intensity contrast [23]. Cell compositional state variables, defined by bond‐specific vibrational contrast and chemical distribution, report endogenous molecular composition. These variables are typically probed using vibrational spectroscopy platforms such as Raman spectroscopy and mid‐infrared (mid‐IR) absorption, which excite molecular bonds with laser light and detect scattered or absorbed photons to recover chemical composition without labels [24]. Cell dielectric state variables, principally specific membrane capacitance and cytoplasm conductivity, report the electrochemical architecture of cellular compartments. These variables are measured by electrical platforms such as impedance cytometry, electrorotation, and dielectrophoresis (DEP), which apply alternating current fields through microelectrodes and measure frequency‐dependent impedance changes as cells polarize in the suspending medium [25, 26]. Because each category operates under distinct physical constraints, their error structures, throughput limits, and specificity trade‐offs differ fundamentally, precluding comparison through a single modality‐agnostic performance metric.

Each of these modality families has undergone substantial development over the past decade. QPI and holotomography have matured from specialized interferometric setups into volumetric subcellular analysis tools capable of resolving organelle‐level RI distributions in three dimensions (3D), though reconstruction stability and computational burden limit throughput under repeated acquisition [27]. Coherent Raman scattering techniques, including stimulated Raman scattering (SRS) and coherent anti‐Stokes Raman scattering (CARS), reduce the throughput gap between spontaneous Raman readout and flow cytometry by accelerating vibrational contrast acquisition [28], with Fourier‐transform CARS (FT‐CARS) implementations further extending broadband chemical discrimination into the fingerprint region. Event rates vary with spectral coverage and with how an event is defined, and implementations that retain broadband fingerprint information typically operate more slowly than approaches that quantify only a limited set of spectral bands. Mid‐IR photothermal imaging (MIP) has emerged as a complementary vibrational modality that exploits the intrinsically larger absorption cross‐section of mid‐IR transitions to achieve video‐rate chemical imaging of live cells [29, 30]. Both vibrational approaches, however, face trade‐offs between chemical breadth and acquisition rate under stable baseline control [31]. In parallel, microfluidic impedance cytometry has advanced from simple two‐frequency sizing to broadband dielectric spectroscopy and physics‐informed real‐time inversion, enabling the extraction of intrinsic membrane and cytoplasmic properties at throughputs exceeding 10^5^ cells per hour [32]. Electrorotation and dielectrophoretic profiling provide complementary routes to dielectric characterization through torque‐driven motion and force‐balance measurements, respectively [33, 34]. Electrical platforms achieve high event rates without light exposure, yet their spatially integrated readouts remain model‐dependent, shifting the burden toward electrode interface stability, medium conductivity control, and identifiability of inverse mappings [35]. Despite the growing importance of label‐free single‐cell monitoring and substantial progress within individual modalities, no prior review has provided a comprehensive framework that systematically links measurement physics to the corresponding cellular state variables. It also remains unclear how measured cell features relate to the analytical requirements for longitudinal inference under repeated acquisition. Cross‐modal comparison is further hindered by inconsistent validation references and non‐aligned performance metrics, making it difficult to determine whether reported descriptors remain stable over time [36]. As a result, existing platforms are not organized in a way that clearly connects how a measurement works to what cellular state it actually reports, nor do they clearly distinguish true monitoring from one‐time profiling [37].

**FIGURE 1:**
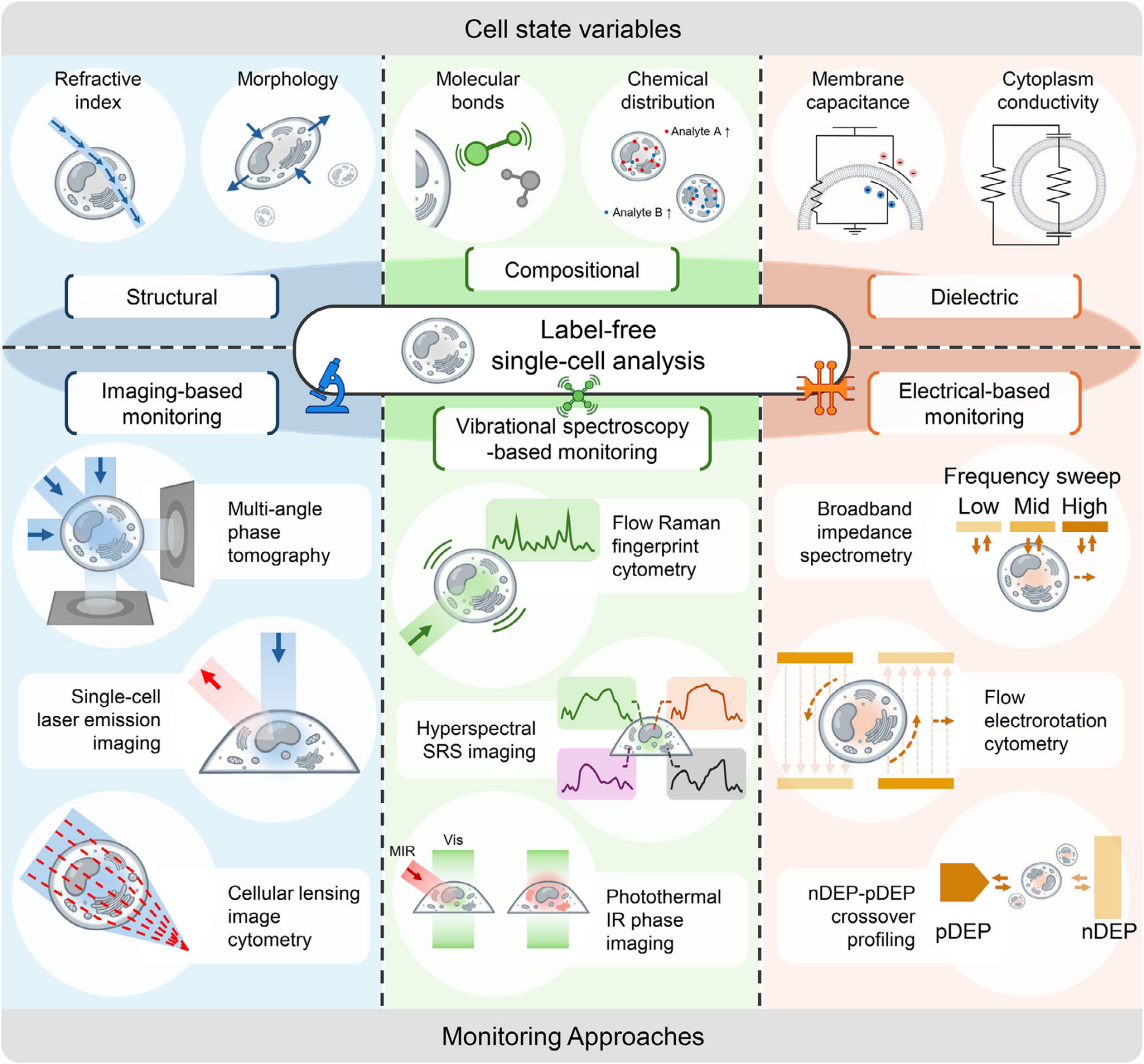
Schematic overview of the analytical mechanisms, including imaging‐, vibrational spectroscopy‐, and electrical sensing‐based label‐free single‐cell monitoring.

In this review, we outline a comparative framework for label‐free single‐cell monitoring that organizes three physically distinct measurement families, including imaging‐based, vibrational spectroscopy‐based, and electrical sensing‐based, according to the cell state variables each family reports (Figure 1). The three modalities are reviewed in a sequence that traces how distinct sensing interfaces translate cellular state into different feature spaces, and a common comparative frame spanning physical principle, workflow design, feature definition, quantitative performance, validation practice, and implementation constraints is applied throughout so that cross‐modal comparisons remain anchored to monitoring requirements [38]. In the outlook, we identify cross‐modal design and reporting needs, including calibration standards, stability metrics, and interface robustness that define the analytical requirements for reproducible longitudinal single‐cell monitoring [39]. Longitudinal monitoring in this review represents repeated measurements acquired under defined conditions and along a defined temporal axis, with extracted features remaining quantitatively comparable across acquisitions and observed shifts attributed to biological rather than instrumental origins [40, 41]. This definition is used throughout the following sections to indicate whether each platform directly demonstrates longitudinal monitoring or provides a more limited time‐ordered application.

## 2 Imaging‐Based Single‐Cell Monitoring Platforms for Label‐Free Structural Characterizations

Imaging‐based label‐free monitoring recovers cell structural state by measuring changes in a coherent light beam as it passes through intracellular material, including membranes, organelles, and nucleic acids, each with an RI that differs from that of the surrounding medium [42]. These interactions impose spatially patterned changes in the phase and direction of the transmitted wavefront, encoding information about the cell's internal structure. Spatial information within the cell is preserved in each measurement, allowing structural changes in specific compartments such as the nucleus, cytoplasm, and organelles to be distinguished and quantified by location [43]. Furthermore, cell boundary‐derived morphological information and intracellular structural information can be acquired simultaneously within the same imaging coordinate system, enabling morphological and internal structural changes to be interpreted in relation to each other. However, RI contrast reflects contributions from multiple molecular components, so structurally similar phenotypes can represent distinct biological states, and consistent control of focus position, illumination uniformity, and registration conditions is required to ensure reproducibility [44, 45]. Because longitudinal imaging generates large numbers of frames and robust interpretation depends on comparing subtle RI‐contrast differences across time points and cell populations, automated analysis, including machine learning‐based inference, is increasingly applied to support consistent and scalable quantification. These properties make imaging‐based platforms applicable to contexts where spatially resolved structural readouts are needed, including drug response dynamics, cell cycle progression analysis, phenotypic heterogeneity quantification in clinical samples, and quality monitoring in cell manufacturing [46]. From a practical sample preparation perspective, the four imaging platforms separate according to whether cells must be stabilized on a surface before measurement. Holotomography follows cells maintained in adherent culture on glass during time‐lapse imaging, whereas SLEC, DLIC, and NCC interrogate cells introduced in suspension under flow or within a planar cavity, avoiding a prior attachment step. Imaging‐based monitoring platforms are distinguished by how much spatial structural information they preserve during feature extraction. Holotomography retains the 3D RI field for broad structural phenotyping but requires substantial reconstruction. SLEC compresses nucleolus‐linked structure into a laser fingerprint, DLIC reduces the readout to size, eccentricity, and average RI under flow, and NCC extends the readout into chemical efflux through cellular lensing and nanosensor transduction. Resonant waveguide grating imaging and imaging surface plasmon resonance microscopy extend this structural monitoring framework to adherent cell geometries by probing cell‐substrate adhesion dynamics and membrane‐proximal refractive index changes at the single‐cell level [47, 48, 49], though longitudinal comparability in these configurations depends on surface regeneration stability and consistent cell‐substrate contact conditions.

### 2.1 3D Holotomography

Holotomography reconstructs a 3D RI distribution from multiplexed phase‐sensitive measurements acquired under structured illumination, providing volumetric subcellular contrast that supports longitudinal monitoring without fluorescent labels [50]. Quantitative phase and RI‐driven contrast enable repeated observation when fluorescence would otherwise impose delivery, bleaching, and phototoxicity constraints [51]. The implementation discussed here uses four optimized illumination wavefronts with computational deconvolution to recover the volume [52, 53]. Reconstructed volumes can be distilled into two‐dimensional (2D) MIP images to support faster inference, trading full 3D context for a lower‐cost representation that still captures dominant morphology. Holotomography provides a physically interpretable coordinate anchored in optical path delay, and the volumetric readout preserves subcellular context that is essential for separating structurally overlapping phenotypes such as apoptosis, necroptosis, and necrosis. However, RI‐based inference remains indirect, so mechanistic attribution can require compositional or functional complements when distinct biological programs collapse onto similar structural signatures [54]. Calibration and reconstruction settings, together with projection choices, can shift the measured contrast in repeatable ways that propagate into learned representations and complicate biological attribution across cohorts [55]. Projection‐based inference improves speed but can obscure localized precursors when early pathway cues are confined to subcellular regions that do not survive axial compression [56].

**FIGURE 2:**
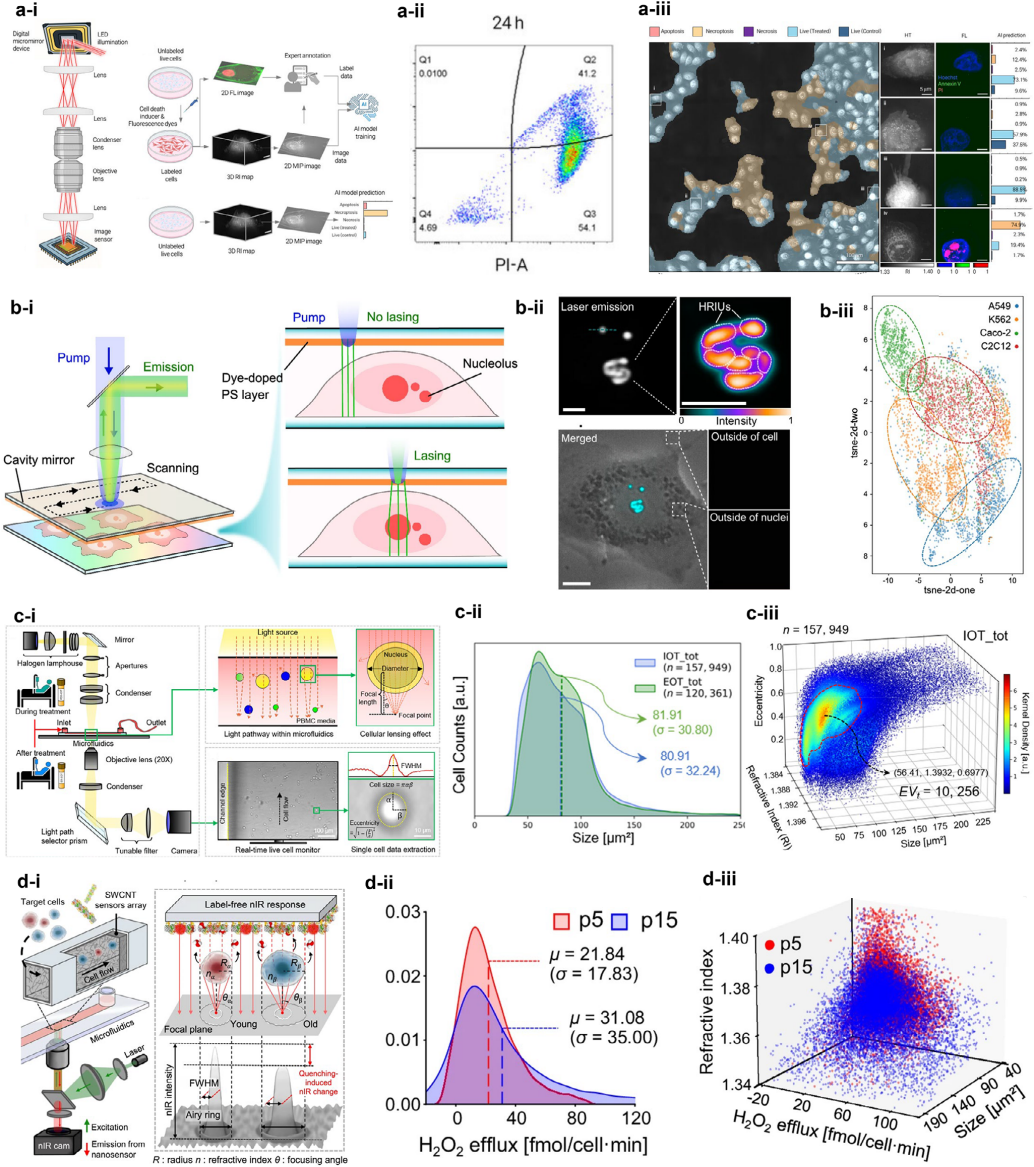
Imaging‐based label‐free single‐cell monitoring platforms. (a) Holotomography platform for label‐free monitoring of cell‐death pathways: (i) schematics of 3D holotomography and deep learning, (ii) cytometry quadrant plot (Annexin V vs PI), and (iii) patch‐wise prediction map on an RI MIP image. Reproduced under the terms of the CC‐BY license [57]. Copyright 2026, Kim et al., published by Wiley‐VCH GmbH. (b) SLEC platform for label‐free nucleolus fingerprinting: (i) schematics of optical cavity readout, (ii) laser‐emission spots and emission‐intensity map, and (iii) t‐SNE projection of single‐cell emission fingerprints. Reproduced with permission [65]. Copyright 2024, Fang et al., published by Springer Nature. (c) DLIC platform for profiling PBMCs during treatment: (i) schematics of the DLIC setup and workflow based on cellular lensing, (ii) distribution curves of single‐cell size at IOT and EOT, and (iii) 3D correlation map of cell size, RI, and eccentricity. Reproduced under the terms of the CC‐BY license [73]. Copyright 2024, Park et al., published by John Wiley & Sons Australia, Ltd. (d) NCC platform for quantitative analysis of H_2_O_2_ efflux in heterogeneously aging human dermal fibroblasts: (i) schematics of the NCC single‐cell monitoring mechanism, (ii) distribution curves of cell size in p5 and p15 fibroblasts, and (iii) 3D correlation map of cell size, H_2_O_2_ efflux, and RI. Reproduced with permission [84]. Copyright 2025, Song et al., published by Springer Nature.

Kim et al. developed a real‐time label‐free workflow that combined time‐lapse 3D holotomography with a convolutional neural network (CNN)‐based inference for classification of apoptosis, necroptosis, and necrosis (Figure 2a) [57]. In their workflow, 3D RI tomograms were converted into 2D MIP representations for model input, while coregistered fluorescence channels provided the reference labels used for supervised training (Figure 2a‐i). This projection‐based representation enabled rapid inference under dense culture conditions. Time‐lapse datasets were acquired at 30‐min intervals over 24 h, and transfer learning with ResNet‐101 on projection images maintained performance at confluency up to 70%. Volumetric RI renderings supported interpretation by revealing pathway‐dependent shifts in RI heterogeneity and concurrent shape changes (Figure 2a‐ii). ResNet‐101 operates as a data‐driven classifier whose decision boundaries are not directly tied to physically interpretable RI features, so biological attribution of individual predictions requires coregistered fluorescence ground truth rather than being derivable from the model output alone. The 3D approach was especially important for drug‐induced phenotype classification, where 2D surrogate representations showed substantially weaker discrimination. Label‐free trajectory predictions were validated by registering fluorescence assays as a reference, enabling cross‐checking of temporal alignment between intrinsic RI cues and biochemical markers (Figure 2a‐iii). The model predicted the necroptotic transition approximately two to 4 h earlier than conventional fluorescence markers such as Annexin V and propidium iodide [58], which underscores both the potential lead time of intrinsic cues and the importance of defining a validation criterion around temporal ordering and sustained separation during progression. Cross‐cell‐line generalizability improved after fine‐tuning, indicating practical transferability across biological backgrounds. The holotomography workflow provides repeated 24 h time‐lapse measurements and therefore demonstrates longitudinal monitoring within a defined cell‐death trajectory.

### 2.2 Single‐Cell Laser Emitting Cytometry

Single‐cell laser emitting cytometry (SLEC) embeds living cells in a planar optical resonator and reads out stimulated emission as a single‐cell signature by concentrating nucleolus‐centered RI contrast into a bright emission fingerprint [59]. SLEC is built around a Fabry‐Perot microcavity formed by two dielectric mirrors [60]. An extracellular gain layer, such as a dye‐doped polymer film, enables optical pumping without intracellular labels and enhances threshold contrast between the nucleolus and surrounding regions [61]. Each cell typically produces multiple distinct lasing peaks, whose spectral positions and intensities encode nucleolar geometry and size, providing a cell‐specific emission fingerprint with high spectral resolution. Unlike holotomography, which preserves a continuous RI field for broad structural phenotyping, SLEC compresses the intracellular state into a threshold‐mediated fingerprint that increases sensitivity to nucleolus‐linked structure [62]. Quantification parameterizes the emission output into low‐dimensional descriptors that combine spatial pattern metrics with spectral peak statistics, where peak number within a free spectral range maps to nucleolus number and peak intensity scales with nucleolus size [63]. The cell itself remains unlabeled, but the burden of exogenous chemistry is shifted to the cavity through the dye‐doped gain layer, which becomes a stability and aging variable of the instrument [64]. Longitudinal comparability, therefore, depends on maintaining cavity alignment and pump conditions across sessions, and dye photobleaching and gain layer lifetime remain practical limits.

Fang et al. developed an end‐to‐end acquisition scheme in which cells are interrogated within the microcavity, and both emission images and spectra are recorded as a coupled fingerprint (Figure 2b) [65, 66]. Cells can be positioned in an array format for repeated interrogation or delivered through a cavity‐integrated microfluidic channel for event‐by‐event readout. A scanned pump beam interrogates cells within the Fabry‐Perot cavity and selectively triggers nucleolus‐localized lasing once the threshold condition is met, so event timing is defined by the pump and imaging frame interval rather than by passive passage (Figure 2b‐i). The spatial and spectral descriptors can be extracted per cell and compared across populations under matched cavity and pump settings (Figure 2b‐ii). The spatial laser emission mode and the emission spectrum capture complementary information from the cell, so combining these two readouts supports more robust comparisons. A second quantitative axis is the lasing threshold, which varies with nucleolus‐linked structure and provides an additional physically interpretable parameter when the pump window is explicitly defined. Population‐level embeddings were used to support monitoring‐relevant mapping between fingerprint structure and subcellular architecture (Figure 2b‐iii) [67]. In the flow‐compatible configuration, throughput reaches cytometry scale with reported event rates up to 2,000 events per second after hardware upgrades, and the platform has been extended to tissue sections [68], where emission fingerprints supported label‐free discrimination of tumor versus healthy regions consistent with histological references. SLEC supports repeated optical interrogation and monitoring‐relevant descriptor extraction, but direct longitudinal monitoring along a defined biological time axis has not yet been shown.

### 2.3 Bright‐Field Cellular Lensing Cytometry

Deep learning‐enhanced image cytometry (DLIC) extracts intrinsic biophysical phenotypes from standard bright‐field image streams in a simple microfluidic format, producing cohort‐scale readouts without staining or genetic modification. Contrast is derived from how cells reshape transmitted illumination rather than from tag‐dependent specificity. When visible or near‐infrared (nIR) light passes through a suspended cell whose RI differs modestly from the surrounding medium, the cell behaves as a weak lens and forms a focused high‐intensity feature on the shadow side, often termed a photonic nanojet [69, 70, 71]. A physics‐based mapping converts this nanojet intensity profile into an average RI estimate, while contours from major and minor axes provide size and eccentricity. This approach occupies an intermediate point between holotomography and SLEC by retaining an RI‐grounded coordinate without volumetric reconstruction and by using a compact feature set for label‐free monitoring. By restricting the feature set to parameters measurable with a conventional microscope, DLIC requires no additional image processing or staining steps, enabling rapid and operationally simple measurements that are compatible with on‐site and online deployment. However, the RI estimate is only as stable as the nanojet feature under focus drift, lamp spectrum shifts, and channel optics changes, which necessitates explicit control of acquisition setpoints and event‐quality rules. Changes in illumination spectrum, channel optics, or cell geometry outside the simulation priors can bias RI mapping and reshape downstream distributions even when underlying biology is unchanged [72]. The simulation‐calibrated physical model can overfit to its training domain and modeling assumptions, so generalizability to new cell types or acquisition conditions requires revalidation.

Park et al. developed a pipeline that maps bright‐field frames to per‐cell biophysical coordinates under microfluidic flow, where a deep learning detector localizes cells in channel images and a finite‐difference time‐domain (FDTD) simulation converts nanojet profiles into RI estimates (Figure 2c) [73]. Cell localization is the deep learning contribution, whereas RI estimation remains tied to an explicit physical mapping initialized using FDTD simulations under representative geometries. The two components differ in interpretability because the FDTD‐calibrated RI output can be independently verified against known physical standards, whereas the localization model depends on the training distribution and therefore requires revalidation for new cell types or channel geometries. This pipeline was applied to characterize PBMC biophysical heterogeneity in patients with extranodal NK/T cell lymphoma, analyzing more than 270, 000 single cells across serial treatment stages (Figure 2c‐i). Distribution‐level comparisons were presented that treat cohort shifts as changes in population structure rather than as changes in a small set of labeled subtypes (Figure 2c‐ii). Single‐cell values for each indicator were summarized as non‐Gaussian distributions, and pairwise density views revealed correlated shifts not apparent from marginals alone, with all three indicators contributing to statistically separable population structure. To quantify contraction or expansion of the biophysical landscape across stages, an effective volume metric was defined by weighing local occupancy as the inverse of kernel density [74]. The resulting metric captured directional contraction of heterogeneity and coordinated shifts in morphology‐linked indicators within a 3D state cloud over size, eccentricity, and RI (Figure 2c‐iii). These results suggest that label‐free biophysical coordinates derived from standard bright‐field frames can serve as a practical monitoring tool for tracking treatment‐induced population shifts in clinical blood samples without staining or specialized optics. DLIC resolves treatment‐stage–ordered cohort shifts in a shared biophysical coordinate system, but it does not provide repeated longitudinal measurements of the same cells.

### 2.4 Nanosensor Chemical Cytometry

Nanosensor chemical cytometry (NCC) couples an optical nanosensor interface with microfluidic measurement to acquire extracellular chemical efflux trajectories from individual cells while maintaining time‐registered biophysical descriptors [75]. NCC separates sensing and registration into two coupled layers, in which a stationary nanosensor array provides analyte‐selective transduction and cellular lensing assigns each time trace to a specific passing cell. Photonic nanojet lensing is favored when the ratio of the cell RI to the suspending‐medium RI is below two and when the cell diameter falls within a window spanning two to forty times the emission wavelength. Under these optical conditions, nIR fluorescence from the nanosensor array can form a localized downstream intensity maximum that serves as the registration event [76]. SWCNTs are used as the primary sensing layer because their nIR emission is photostable, chemically tunable, and spectrally matched to support nanojet‐based registration at cellular length scales, and other nIR‐emitting materials with comparable photostability and emission wavelength are also compatible with the cellular lensing registration scheme [77, 78]. The sensing axis is set by the choice of SWCNT surface chemistry, and exchangeable molecular recognition elements allow the target space to extend across diverse biochemical efflux targets, including reactive oxygen and nitrogen species, metabolites, and proteins [79, 80, 81]. The nanojet profile varies with intracellular composition and organelle distribution, introducing cell‐state‐dependent variation in the registered optical signature [82]. Unlike imaging‐based platforms that report morphological and RI features, NCC adds a direct chemical quantification axis from the same single‐cell event stream, and transport‐reaction modeling translates nanosensor intensity changes into per‐cell efflux at the attomolar level. The nIR nanosensor readout is photostable, supporting repeated monitoring of chemical dynamics. The same event stream also captures biophysical context, such as size and RI, enabling efflux heterogeneity to be interpreted alongside physical state. However, quantification depends on cell‐to‐array distance, flow timing, and array uniformity, and chemical coverage remains tied to the chosen sensor chemistry unless multiple sensor‐analyte pairs are integrated [83].

Song et al. used NCC to investigate single‐cell extracellular chemical efflux dynamics under controlled microfluidic flow, linking each nanosensor time trace to an individual cell via cellular lensing while specifying analyte selectivity through the sensor chemistry (Figure 2d) [84]. Cells are introduced in suspension and transported through the channel under controlled flow, and recent implementations use deep learning detection for automated event localization with high detection fidelity, supporting practical cohort‐scale throughput on the order of 10^5^ cells per hour (Figure 2d‐i). Deep learning contributes only to event localization, while efflux quantification is governed by a physically derived transport‐reaction model, preserving biochemical interpretability of the primary readout independently of the training data used for detection. A time‐registered readout tracks sensor emission at matched cell coordinates across repeated imaging, enabling per‐cell efflux descriptors to be extracted without intracellular labeling (Figure 2d‐ii). A multivariate representation couples biophysical context with analyte‐linked nanosensor modulation to resolve cohort‐scale shifts, where a joint distribution in the combined biophysical and chemical coordinate system allows population structure to be compared across conditions (Figure 2d‐iii). In aging‐oriented cohorts, this framework resolved coordinated shifts across all four phenotypes with increased heterogeneity at later passages, and derived trajectory representations captured directional expansion of the aging state space. Beyond aging, the same NCC framework is applicable to cell therapy quality assessment, cancer cell heterogeneity profiling, stem cell stemness monitoring, and cosmetic efficacy evaluation. NCC can quantify single‐cell efflux trajectories alongside size and RI features obtained from the same event, enabling analysis that compares efflux distributions and identifies minority subpopulations with distinct efflux magnitude or recovery behavior under matched assay conditions. These applications share a common requirement for simultaneous chemical and biophysical readouts at single‐cell resolution, which neither imaging nor chemical assays can provide independently.

## 3 Vibrational Spectroscopy‐Based Single‐Cell Monitoring Platforms for Label‐Free Compositional Characterizations

Vibrational spectroscopy‐based platforms recover cell compositional state by coupling optical excitation to bond‐specific molecular vibrations, using the resulting spectral fingerprint as a chemically informative readout rather than inferring state from morphological proxies [85]. When optical excitation couples to molecular vibrations, vibrational resonances produce wavelength‐dependent responses that can be captured as Raman‐shifted scattering or as mid‐IR absorption with photothermal detection. The resulting pattern of vibrational bands across the spectrum provides a chemical fingerprint that reflects the relative contributions of proteins, lipids, nucleic acids, and metabolites within the cell [86, 87]. Compositional changes are typically inferred from bond‐level spectral features after background correction and model‐guided processing, providing chemical contrast that complements structural and electrical readouts [88]. In addition, the spectral fingerprint encodes information from multiple molecular classes simultaneously, so coordinated state changes spanning proteins, lipids, and nucleic acids can be captured in a single measurement without targeting individual markers. However, signal levels per cell are inherently limited by the efficiency of the vibrational coupling mechanism, and broadband background signals, including autofluorescence can obscure vibrational features, requiring careful balance among spectral resolution, acquisition time, and light dose to ensure reproducibility [89]. These properties make vibrational spectroscopy‐based platforms applicable to contexts where direct chemical readout is required, including metabolic state tracking, drug‐induced compositional change monitoring, and cell phenotyping based on molecular composition rather than morphology [90]. In the vibrational platforms, handling is determined mainly by how cells are brought to the optical interrogation region rather than by a single common loading format. FT‐CARS and spontaneous Raman flow cytometry introduce suspended cells through microfluidic channels, with acoustic focusing or pDEP‐DLD confinement used to control cell position, whereas hyperspectral SRS relies on fixed‐cell preparation and MIP‐QPI images live cells directly in aqueous conditions.

For vibrational monitoring, the central trade‐off lies among spectral breadth, acquisition speed, and live‐cell compatibility. FT‐CARS enables high‐throughput fingerprint‐region sorting but remains restricted to that spectral window and relies on ultrashort pulses. Spontaneous Raman preserves full‐spectrum coverage with multi‐hour stability but remains throughput‐limited by weak scattering. Hyperspectral SRS provides subcellular chemical mapping, yet is currently fixed‐cell, whereas MIP‐QPI supports video‐rate live‐cell imaging with lower photodamage but mainly in the high‐wavenumber region.

### 3.1 Fourier‐Transform Coherent Anti‐Stokes Raman Flow Cytometry

Coherent Raman flow cytometry defines an event through a flow‐aligned trigger that gates a brief acquisition window and reconstructs a broadband Raman fingerprint from short transits, so the spectrum itself functions as the per‐cell compositional readout without labels [91, 92]. In rapid‐scan FT‐CARS implementations, broadband molecular fingerprints over approximately 300 to 1,600 cm^−1^ can be acquired at rates compatible with flow handling and flow‐based single‐cell acquisition, providing chemistry‐specific discrimination while preserving event‐level throughput [93]. This chemically anchored readout complements structure‐oriented measures such as RI imaging and bright‐field biophysical profiling, because intracellular composition can diverge in bond‐specific spectra even when size or shape changes are subtle. Event rates in the tens of events per second range can be achieved while retaining chemically interpretable fingerprints [94]. However, throughput and chemical coverage remain coupled. Faster operation shortens the time available to accumulate signal within each event, which can reduce spectral stability. Broader spectral coverage increases acquisition time and computation, which can delay real‐time spectral stability and increase variability across cells. Beyond this acquisition‐coverage trade‐off, spectral intensity can drift across days, and high excitation powers can introduce photodamage and baseline distortions that can be misread as biochemical variation. Event definition, calibration, baseline handling, and normalization therefore, become part of the reported metrology rather than implicit processing choices. Media background, dose constraints, and partial sampling during short transits can further increase variance, making explicit reporting of event definition and calibration essential for cross‐session comparability [95].

**FIGURE 3:**
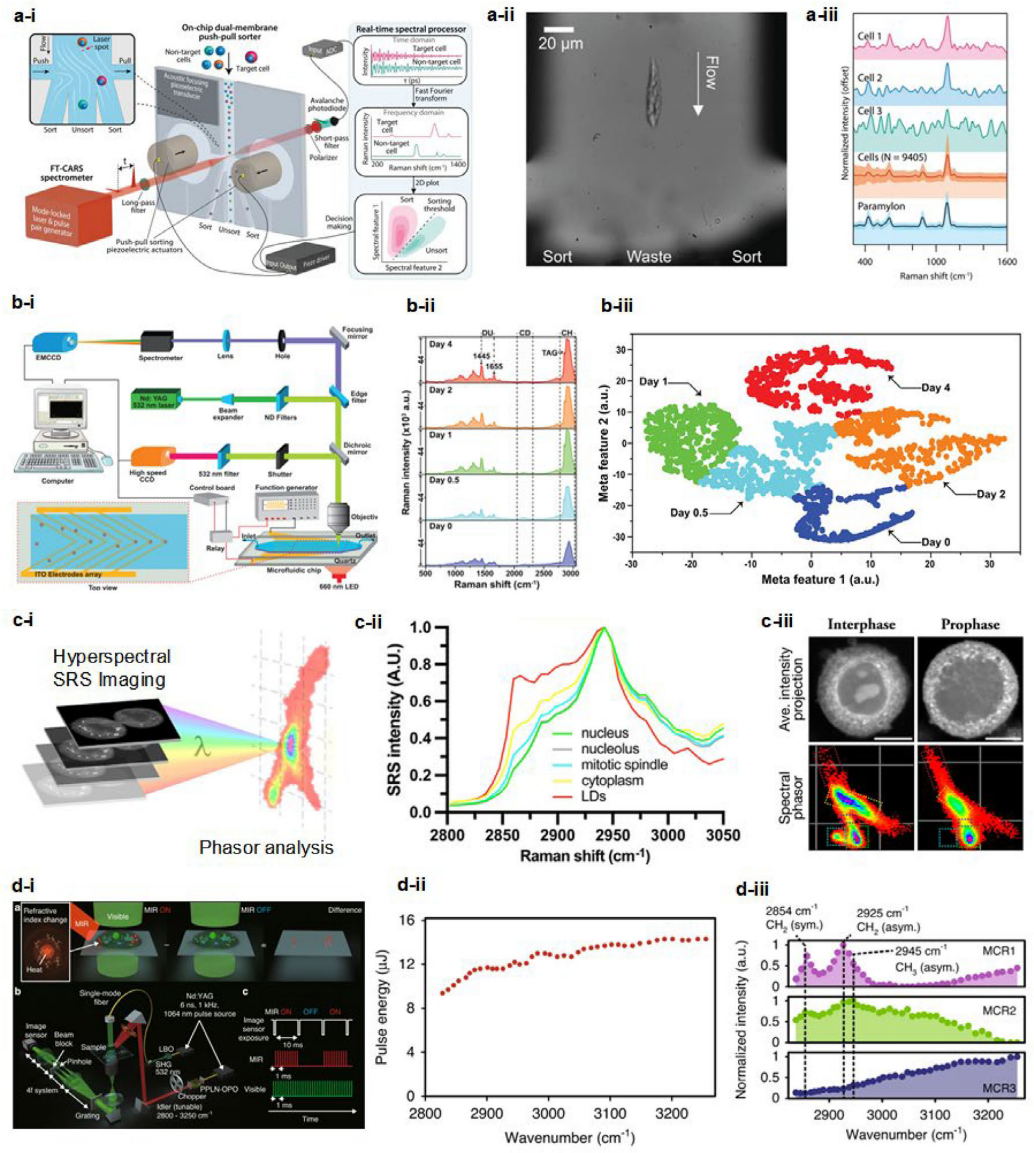
Vibrational spectroscopy‐based label‐free single‐cell monitoring platforms. (a) FT‐CARS Raman‐activated cell sorting in the fingerprint region: (i) platform schematic linking FT‐CARS readout to an on‐chip push–pull sorter with real‐time processing, (ii) bright‐field sequence through the measurement and sorting regions, and (iii) representative single‐cell fingerprint spectra with an ensemble reference. Reproduced with permission [96]. Copyright 2022, Lindley et al., Wiley‐VCH. (b) Spontaneous Raman flow cytometry platform for single‐cell metabolic phenome profiling: (i) schematics of the pDEP–DLD–RFC system integrated on a microfluidic chip, (ii) time‐course Raman spectra highlighting band‐level metabolic shifts, and (iii) t‐SNE map of single‐cell spectral features. Reproduced under the terms of the CC‐BY license [98]. Copyright 2023, Wang et al., published by Wiley‐VCH GmbH. (c) Hyperspectral SRS imaging platform with spectral phasor analysis for mitosis profiling: (i) schematics of hyperspectral SRS imaging and spectral phasor analysis, (ii) organelle‐associated SRS spectra in the high‐wavenumber region, and (iii) average intensity projection and spectral phasor maps of interphase and prophase cells. Reproduced under the terms of the CC‐BY license [109]. Copyright 2023, Hislop et al., American Chemical Society. (d) MIP–QPI platform for live‐cell vibrational imaging: (i) schematics of the wide‐field MIP–QPI system and MIR‐ON/OFF differential measurement, (ii) MIR pulse energy across the tuning range, and (iii) MCR components with bands in the high‐wavenumber region. Reproduced under the terms of the CC‐BY license [115]. Copyright 2023, Ishigane et al., published by Springer Nature.

Lindley et al. combined on‐chip acoustic focusing with an upstream forward‐scatter trigger and a Fourier transform CARS readout so that each cell could be intercepted under continuous flow and assigned a fingerprint‐region spectrum within a gated acquisition window (Figure 3a) [96]. Acoustic focusing reduced trajectory dispersion, and forward‐scatter triggering defined a reproducible event time stamp before the cell entered the CARS focus, strengthening measurement consistency across cells during streaming operation (Figure 3a‐i). The two‐region layout further imposed a fixed transit from measurement to actuation, and bright‐field images captured cells as they moved from the measurement region to the sorting junction (Figure 3a‐ii). Time‐domain interferometric traces were Fourier transformed in real time to generate event‐resolved Raman spectra. Measurements from polymer microbeads and multiple microalgal models were reported alongside standard samples of paramylon, starch, and astaxanthin, allowing paramylon‐associated bands to be interpreted under a common normalization framework (Figure 3a‐iii). A population‐level chemical boundary was defined from a non‐target reference distribution and applied during mixed‐sample operation, discriminating paramylon‐rich Euglena gracilis cells from paramylon‐poor phenotypes using the intensity of a characteristic Raman peak. Chlorophyll autofluorescence imaging of the separated fractions provided an orthogonal reference that confirmed the Raman‐defined spectral boundary against an independent photophysical marker, validating that the per‐cell compositional readout remained chemically interpretable under continuous flow conditions. At a throughput in the tens of events per second, spectral discrimination fidelity exceeded 90% under a strict chemical gate, reflecting the inherent trade‐off between discrimination stringency and population recovery under mixed‐population conditions. Gate criteria, decision thresholds, and external validation checks are therefore necessary reporting components alongside throughput metrics for cross‐session comparability [95]. FT‐CARS functions as a real‐time event‐resolved sorting platform, whereas repeated measurements along a biological time axis were not part of the demonstration.

### 3.2 Spontaneous Raman Flow Cytometry

Spontaneous Raman flow cytometry acquires a full biochemical spectrum around 550–3,100 cm^−^^1^ per cell by collecting inelastically scattered photons within a controlled interrogation window during flow, preserving broad chemical information while supporting event‐based processing. The advantage is chemical breadth without labels, but the weak scattering cross‐section couples acquisition time, photon dose, and event definition, so monitoring performance depends on whether interrogation geometry and baseline remain stationary over long runs. A practical strategy is to stabilize interrogation by combining spontaneous Raman detection with electrokinetic control that converts uncertain transits into repeatable events [97]. Dielectrophoretic trapping can immobilize cells at the laser spot without optical trapping, achieving trapping efficiencies above 90% under stated electrical conditions. Stable operation extends to approximately 5 h with throughput spanning roughly 30 to 2, 700 events per minute, depending on acquisition and recording settings [98]. However, electrical positioning depends on medium conductivity, temperature, and electrode geometry, and operation can introduce Joule heating that affects both baseline and physiology. Full‐spectrum acquisition preserves chemical breadth but keeps speed and dose coupled, and electrode material and drive settings become part of optical baseline control under visible excitation.

Wang et al. developed a positive dielectrophoresis‐deterministic lateral displacement (pDEP‐DLD) layout that converts flow variability into timing‐controlled interrogation at a fixed region, where event acceptance is engineered upstream through electrokinetic control rather than inferred downstream from spectra alone (Figure 3b) [98, 99]. The pDEP‐DLD‐RFC setup integrates a Raman microscope with a microfluidic chip bearing a sloped Indium tin oxide electrode array, where relay‐timed pDEP actuation synchronizes single‐cell positioning with the spectral acquisition window (Figure 3b‐i). Tight coupling between focusing and triggering thereby aligned short acquisition windows with flowing single‐cell transits. Reduction from full spectra to monitoring descriptors was demonstrated using band‐integrated metrics for lipid‐associated CH features, unsaturation‐linked signatures, and isotope channels when metabolic flux is probed with deuterated substrates (Figure 3b‐ii) [100]. Learned embeddings compressed spectra into low‐dimensional coordinates while preserving multiband covariation, and the same framework supported metabolically distinct profiling across microalgae, yeast, bacteria, and human cancer cell lines, indicating broad transferability of the event‐controlled spontaneous Raman workflow (Figure 3b‐iii). The CNN classifier provides data‐driven spectral discrimination whose attribution depends on the training distribution, whereas intra‐ramanome correlation analysis retains direct biochemical interpretability through bond‐specific spectral assignments, representing two distinct levels of model interpretability within the same platform. Monitoring performance was summarized through distribution‐level stability checks that accompany spectral separation, consistent with multi‐hour acquisition, where baseline stationarity and acceptance rate dominate error. Correlation‐network analyses of intra‐ramanome structure further exposed condition‐specific metabolite‐coupling patterns, extending interpretation beyond endpoint class labels [101, 102]. Spontaneous Raman flow cytometry captures cohort‐level temporal changes in astaxanthin accumulation and lipid dynamics, but the same cells were not measured repeatedly across time.

### 3.3 Hyperspectral Stimulated Raman Microscopy With Spectral Phasor Analysis

Hyperspectral SRS microscopy with spectral phasor compression combines label‐free vibrational contrast with compartment‐level spatial context [103]. Images are acquired at multiple Raman shifts, giving each pixel a short spectrum that forms a hyperspectral image stack. Spectral phasor analysis maps each pixel spectrum onto a 2D plane using Fourier components, where mixtures and dominant components form separable groupings without explicit peak fitting or a trained model [104, 105]. The defining event is not a whole‐cell transit but a per‐cell set of compartment‐resolved pixel ensembles whose spectra are summarized in a common phasor space. Chemical descriptors are anchored to a segmented compartment, such as nucleus, cytoplasm, or lipid droplets, which reduces ambiguity from intracellular mixing that confounds whole‐cell fingerprints. In the high‐wavenumber region, lipid‐rich structures emphasize CH_2_‐dominated features, protein‐rich regions contribute stronger CH_3_ bands, and nuclear spectra contain a distinct contribution associated with DNA‐rich content near 2,970 cm^−1^ [106]. However, restricting measurements to a high‐wavenumber window improves speed but reduces chemical specificity. Segmentation quality becomes part of the measurement, phasor cluster selection can be manual, and the effective throughput is set by scan time and photodose constraints [107]. In this configuration, cells are fixed to stabilize biochemistry during acquisition, so the output represents phase‐resolved snapshots rather than continuous live trajectories.

Hislop et al. acquired a multiwavelength image stack across the high‐wavenumber window of 2,800 to 3,050 cm^−1^ by stepping excitation between frames to build a hyperspectral set (Figure 3c) [108, 109]. Pixel spectra were transformed into phasor coordinates, clusters were selected on the phasor plane, and those selections were projected back into the image to generate compartment masks for nucleus, nucleoli, lipid droplets, cytoplasm, and spindle‐associated regions (Figure 3c‐i). PFA‐fixed SK‐BR‐3 cells were staged across interphase plus five mitotic phases with multiple biological replicates per phase [110]. Detectable phase‐dependent modulation in compartment‐anchored signals was reported, including a significant change in the nuclear DNA‐associated vibrational band between prometaphase and metaphase, supported across biological replicates (Figure 3c‐ii). Quantification was expressed as compartment‐specific intensity changes at selected bands and as ratios that normalize within‐spectrum variability, such as 2,970/2,930 in the nucleus and 2,851/2,930 in the cytoplasm. Nuclear spectral coordinates and derived ratios were shown to occupy separable regions across mitotic phases under the same acquisition and masking rules (Figure 3c‐iii). Statistical testing across phases confirmed that observed separations are reproducible across biological repeats rather than an artifact of a single phasor gating outcome. Because the current hyperspectral SRS implementation relies on fixed‐cell snapshots, it does not provide a direct demonstration of longitudinal monitoring.

### 3.4 Mid‐Infrared Photothermal Quantitative Phase Imaging

MIP uses mid‐IR absorption to generate chemical contrast and a visible probe to read out the resulting photothermal response [111]. This separation preserves absorption‐based chemical selectivity while retaining the spatial resolution of the visible probe, supporting live‐cell measurements in aqueous environments. A tunable mid‐IR pump is set to an absorption band so that absorbed energy produces a transient temperature rise that perturbs local RI and optical path length [112, 113]. A visible probe beam reads the photothermal modulation as a phase shift measured by QPI. Nanosecond‐scale MIR pulses and kilohertz repetition rates suppress thermal pile‐up while preserving near‐diffraction‐limited contrast in the differential phase channel. In contrast to SRS, which measures Raman‐active vibrations through coherent nonlinear scattering, MIP probes complementary IR‐active transitions through linear absorption transduced into a photothermal phase signal. Raman‐active and IR‐active modes follow different selection rules, so SRS and MIP provide complementary vibrational contrast and can highlight different functional groups. Compared with SRS, MIP supports wide‐field acquisition that reduces reliance on point‐by‐point raster scanning, enabling parallel monitoring of many cells, and the visible probe simultaneously yields a static phase map that registers structural context and chemical contrast in the same field of view without separate instrumentation [114]. However, water absorption and thermal diffusion introduce a background that SRS does not face to the same degree, and phase stability and thermal boundary conditions become central to assay design, requiring consistent chamber geometry and modulation timing across runs to maintain comparability.

Ishigane et al. designed a wide‐field instrument that uses nanosecond mid‐IR pulses at kilohertz repetition rates together with a QPI camera that alternates mid‐IR on and off frames through synchronized modulation, yielding a single‐band MIP image as a differential phase map (Figure 3d) [115, 116]. A wide‐field QPI camera records alternating frames with the mid‐IR pump switched on and off, and subtraction of each on‐off pair yields a differential phase image that isolates the photothermal response under synchronized pump and probe timing (Figure 3d‐i). High dynamic range image sensors stabilized the differential phase readout by avoiding saturation under strong photothermal modulation. In a high‐speed configuration, single‐band wide‐field MIP achieved video‐rate sampling at 50 frames per second while preserving intracellular contrast. Multi‐wavenumber acquisition scanned 40 bands across 2,800 to 3,250 cm^−1^ and used multivariate curve resolution (MCR) to recover component spectra and spatial weights, with mid‐IR pulse energy characterized over the same range to guide band selection for multi‐band scans (Figure 3d‐ii). Components corresponding to lipids, proteins, and water were separated with distinct spatial localization patterns, enabling compact per‐cell chemical coordinates anchored to endogenous absorption rather than labels. Per‐cell component amplitudes were presented as compact state variables for distribution‐based monitoring, where population density shifts across multivariate space indicate condition‐dependent changes (Figure 3d‐iii) [117]. This workflow yields a feature space intermediate between event‐spectrum Raman cytometry and hyperspectral SRS, because it preserves spatially registered chemical contrast while remaining compatible with wide‐field acquisition. MIP‐QPI provides continuous live‐cell acquisition at video rate and thus constitutes a direct demonstration of longitudinal monitoring.

## 4 Electrical Sensing‐Based Single‐Cell Monitoring Platforms for Label‐Free Dielectric Characterizations

Electrical measurement platforms recover cell dielectric state by applying frequency‐dependent alternating current (AC) fields through microelectrodes and measuring the electrical response as cells polarize in the suspending medium [118, 119]. Because different cellular compartments, including the membrane, cytoplasm, and nucleus, differ in the dielectric permittivity and ionic conductivity of their constituent materials, their electrical contributions to the measured response vary with frequency, and sweeping across frequencies allows membrane‐dominated and interior‐dominated components to be resolved separately [120]. This frequency‐dependent decomposition enables compartment‐specific dielectric parameters such as specific membrane capacitance and cytoplasmic conductivity to be extracted at high throughput without light exposure, which is compatible with extended longitudinal measurements where light exposure must be minimized [121]. However, the measured signal is sensitive not only to cell electrical state but also to electrode geometry, medium conductivity, and flow conditions, and biological interpretation depends on equivalent circuit or dispersion models whose assumptions introduce uncertainty in parameter extraction [122]. These properties make electrical platforms particularly suited to high‐throughput repeated measurements where compartment‐level dielectric parameters are the target readout, including blood and immune cell profiling, batch‐to‐batch comparisons in cell manufacturing, and drug response monitoring under standardized medium and flow conditions. For the electrical platforms, sample preparation is governed primarily by control of the suspending medium rather than by optical geometry or surface attachment. All four methods operate on cells in suspension, but each requires platform‐specific conductivity conditions and field‐defined measurement settings to preserve viability while maintaining dielectric contrast. The four electrical platforms differ in bandwidth, inversion strategy, and readout compression. Broadband impedance spectroscopy captures richer dielectric information but requires post‐processing and lower‐conductivity media. piRT‐IFC reduces acquisition to two frequencies for real‐time extraction of intrinsic parameters at high throughput. cROT provides contact‐free dielectric readout from rotation spectra, whereas DEP crossover profiling compresses dielectric state into a single crossover frequency for rapid assays.

### 4.1 Broadband Microfluidic Impedance Spectroscopy

Electrical impedance spectroscopy offers a label‐free route to single‐cell monitoring when the state variables of interest are electrical properties tied to membrane structure and intracellular conductivity [123]. The sensing principle is that a cell behaves as a dispersive object in an electrolyte under an applied AC field [124]. At lower frequencies, the membrane acts as a capacitive barrier and the response reflects effective size and membrane capacitance, whereas at higher frequencies the field penetrates and the response becomes more sensitive to cytoplasm conductivity and internal organization [125]. For nucleated cells, additional dispersions can arise from the nucleus, motivating multi‐compartment descriptions that map distinct relaxations to membrane, cytoplasm, and nuclear regions. By measuring a frequency‐dependent response for each cell, impedance spectroscopy separates these contributions with a physical model and supports monitoring of shifts in distributions rather than only mean trends [118]. In a representative high‐frequency implementation, the measurement bandwidth extends from approximately 250 kHz to 550 MHz and supports event‐resolved parameter extraction for nucleated mammalian cells at cytometry‐scale throughput [126]. Single‐frequency impedance cytometry is often sufficient for size gating, yet it mixes membrane polarization and interior conduction into a composite readout that is difficult to interpret. Recovered parameters from broadband fitting are physically interpretable and can be compared across populations when calibration is stable. However, the inversion is reliable only when bandwidth, frequency sampling, and electrode‐related transfer functions keep the model fit identifiable under the chosen flow conditions. Electrode polarization and fouling can drift, high‐frequency operation magnifies bandwidth and parasitic constraints, and positional variability couples to fit uncertainty [127]. An additional practical constraint is the trade‐off between signal‐to‐noise favored by higher‐conductivity media, and dielectric resolution, favored by lower‐conductivity media, which narrows the operating window for robust inversion [128]. These constraints motivate designs that acquire multi‐frequency information within a single transit while preserving calibration stability across repeated measurements.

**FIGURE 4:**
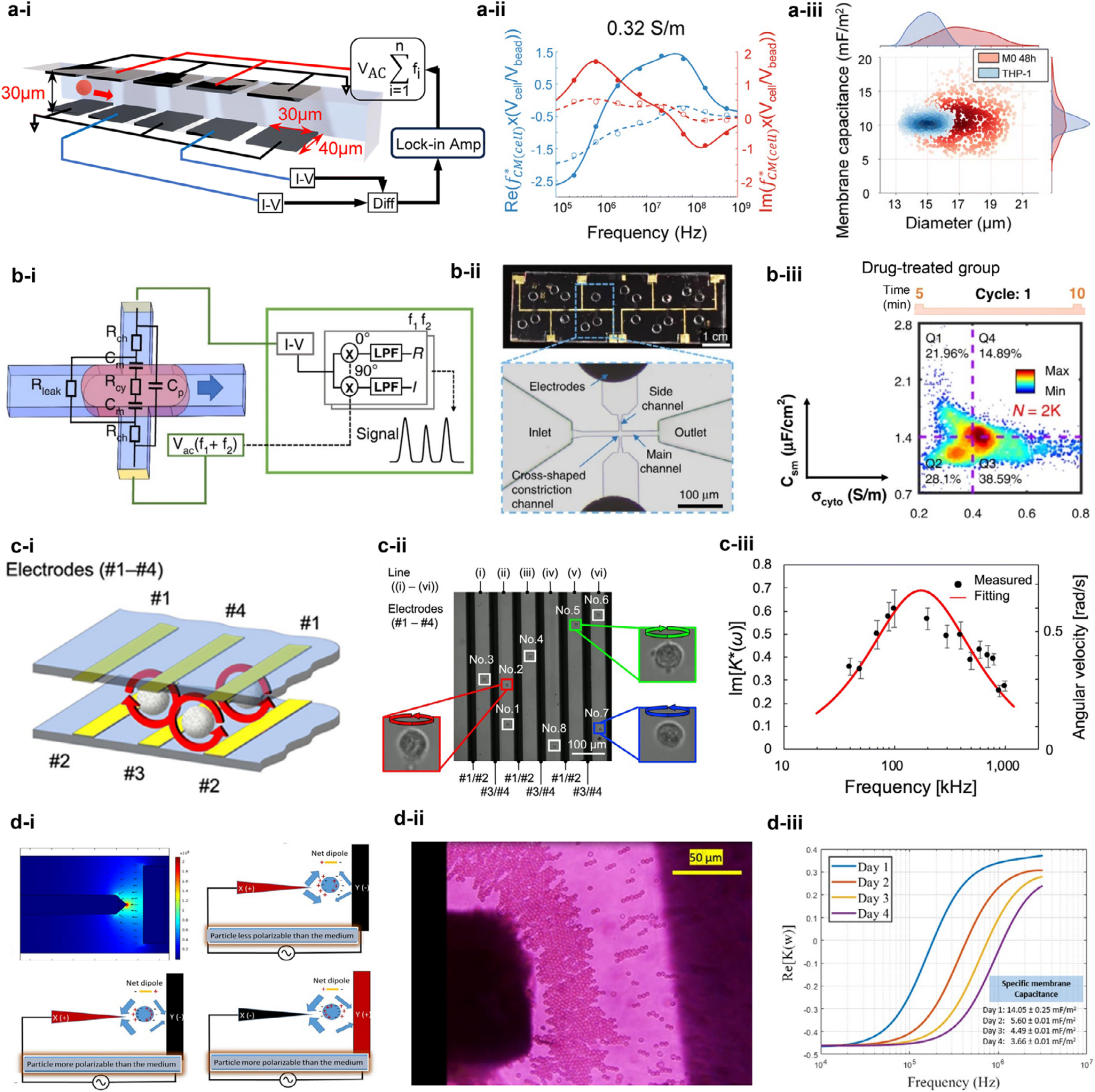
Electrical sensing‐based label‐free single‐cell monitoring platforms. (a) Impedance spectroscopy cytometry for electrical phenotyping of nucleated cells: (i) schematics of a microfluidic impedance cytometer and lock‐in amplifier, (ii) frequency‐dependent impedance spectrum in 0.32 S m^−1^ medium, and (iii) scatter plot of membrane capacitance and diameter between THP‐1 and M0. Reproduced under the terms of the CC‐BY license [129]. Copyright 2025, Zou et al., published by The Royal Society of Chemistry. (b) piRT‐IFC platform for real‐time impedance‐based electrical phenotyping: (i) schematics of the equivalent‐circuit model and two‐frequency impedance readout, (ii) microfluidic chip layout with integrated electrodes and a constriction region, and (iii) scatter plot of single‐cell electrical parameters for a drug‐treated group. Reproduced under the terms of the CC‐BY license [136]. Copyright 2023, Luan et al., published by Springer Nature. (c) Continuous‐flow electrorotation platform for dielectric measurement of single cells: (i) schematics of the electrode configuration generating electrorotation under flow, (ii) field‐of‐view showing multiple measurement positions along the electrode array, and (iii) frequency‐dependent electrorotation response with fitted curve. Reproduced under the terms of the CC‐BY license [142]. Copyright 2023, Yoda et al., published by The Royal Society of Chemistry. (d) DEP platform for label‐free electrical phenotyping of erythrocytes: (i) schematics of positive and negative DEP regimes in a non‐uniform field, (ii) bright‐field image of RBC behavior near the point‐and‐planar device, and (iii) frequency‐dependent DEP response across storage days. Reproduced under the terms of the CC‐BY license [154]. Copyright 2023, Oladokun et al., published by Springer Nature.

Zou et al. implemented a microfluidic impedance platform where multiple discrete frequencies are applied simultaneously to each event, enabling single‐cell spectra to be captured without per‐cell frequency sweeps while preserving throughput (Figure 4a) [129]. A lock‐in chain generates and demodulates an eight‐frequency excitation set within a single transit, and event gating rejects debris, doublets, and off‐axis trajectories because position‐dependent coupling can reshape the spectrum and bias inversion (Figure 4a‐i). Bead‐referenced normalization was shown to convert differential currents into a normalized spectrum proportional to effective polarizability, enabling experimental estimation of a Clausius‐Mossotti‐type response that can be fitted to shell models (Figure 4a‐ii). Reducing medium conductivity from physiological saline (approximately 1.6 S m^−1^) to a lower‐conductivity condition (approximately 0.32 S m^−1^) enhanced dielectric‐relaxation visibility and improved parameter identifiability [130]. Recovered electrical parameters were presented in a scatter representation that highlights separability and within‐population variance, where shifts in central tendency, widening of spread, or emerging tails signal state transitions that a single average would hide (Figure 4a‐iii). Fitting quality was summarized by parameter uncertainty metrics such as coefficient of variation and goodness‐of‐fit, which tightened substantially under optimized conductivity conditions [35]. Broadband impedance spectroscopy places different biological conditions within a common electrical coordinate system, but the reported measurements did not follow a repeated temporal sequence of the same biological process.

### 4.2 Physics‐Informed Real‐Time Impedance Flow Cytometry

Physics‐informed real‐time impedance flow cytometry (piRT‐IFC) reduces the bottleneck of offline per‐cell inversion by pairing measurement with a fast physics‐based solver so that intrinsic parameters are estimated per event during streaming acquisition [131, 132]. Instead of sampling a broadband spectrum, piRT‐IFC measures a dual‐frequency response at 100 kHz and 180 kHz and translates each event into intrinsic electrical properties through an explicit equivalent‐circuit model. This compression is feasible because the microfluidic geometry creates transient high‐resistance sealing when a cell enters a cross‐shaped constriction [133], which increases sensitivity and stabilizes the mapping from raw pulses to cell‐dependent impedance. The main advantage is that the output axis is largely intrinsic and size‐independent, meaning specific membrane capacitance and cytoplasm conductivity can be compared across cohorts without inheriting instrument‐specific impedance units, provided calibration is maintained [134]. However, two‐frequency compression tightens model assumptions, so deviations in sealing quality, leakage pathways, or medium conditions can bias the inferred parameters even when raw pulses meet basic shape and amplitude criteria [135]. Electrode polarization, temperature‐dependent conductivity changes, and surface fouling can shift transfer functions over long experiments, requiring periodic background refresh and quality rules that remove drift without distorting biological distributions.

Luan et al. developed a cross‐shaped constriction with side‐channel geometry where cells experience a reproducible high field and a transient sealing condition as they traverse the sensing zone (Figure 4b) [136]. Transient sealing in the cross‐shaped constriction is represented with an equivalent circuit and interrogated by dual‐frequency lock‐in readout, producing event‐level amplitude and phase features that enable real‐time parameter estimation (Figure 4b‐i). Under consistent sealing, event acceptance is determined primarily by sealing‐dependent impedance changes rather than trajectory scatter, and the constriction‐based sealing and gating logic operates under continuous aspiration flow, so pulses remain stable over long runs. Device photographs and the chip layout document the cross‐shaped constriction and side‐channel geometry that implement this interrogation scheme (Figure 4b‐ii). Real‐time inference was implemented by precomputing circuit responses across a parameter grid on a graphics processing unit (GPU) and caching them as a look‐up table, so per‐cell inference reduces to a fast lookup‐and‐minimization step yielding approximately 0.62 ms per cell [137]. This comparison highlights a clear difference in interpretability between physically grounded and data‐driven approaches. Because the FPPF solver is constrained by an explicit equivalent‐circuit model, it can generalize to biologically unfamiliar cell states, whereas the FCNN predictor loses accuracy on out‐of‐distribution samples because its learned mapping lacks a physical basis for extrapolation. The reported implementation characterizes more than 100,000 cells within a single run while maintaining stable per‐event inference over tens of minutes [132]. Specific membrane capacitance and cytoplasm conductivity were shown to form a 2D map for time‐registered monitoring of distribution shifts under drug treatment (Figure 4b‐iii). Cycle‐to‐cycle similarity quantified with Kullback‐Leibler divergence (KL divergence) remained below 0.1 within the same cell line across cycles while diverging strongly between different cell lines [138]. piRT‐IFC follows distribution shifts over repeated cycles within a defined experimental timeline and therefore directly demonstrates longitudinal monitoring.

### 4.3 Continuous Flow Electrorotation

Continuous‐flow electrorotation converts dielectric dispersion into a rotation response, so the observable becomes an angular velocity that can be compared across runs as a distribution when hydrodynamic and electrical boundary conditions are standardized [139]. Four phase‐shifted AC voltages generate a field whose direction rotates in time, and the induced dipole of a cell lags the field by an amount set by dielectric dispersion. This lag produces an electrical torque that spins the cell until viscous drag balances it, making steady angular velocity proportional to the imaginary component of the Clausius‐Mossotti factor. A single‐shell model maps a frequency‐dependent rotation spectrum to membrane permittivity and cytoplasm conductivity [140], which keeps the recovered coordinates interpretable but constrains identifiability to frequency ranges that sufficiently span the dispersion. Because cells rotate without direct electrical contact to the electrodes, the measurement reduces sensitivity to electrode contact resistance that affects contact‐based impedance readouts. In one continuous‐flow design, interdigitated top and bottom electrode arrays generate a vertical rotation axis while DEP simultaneously aligns cells between electrodes, allowing parallel tracking of multiple cells (up to 32 within a shared observation window) under continuous flow [141]. A demonstrated implementation achieved a measurement throughput of 2,700 cells per hour, placing the method in a cohort‐scale regime compatible with optical tracking. However, angular velocity depends on viscosity and temperature, so long runs require thermal stability and repeated medium checks. Throughput is also limited by the imaging field of view and camera frame rate, and surface adhesion under continuous flow can promote clogging, making surface treatment and channel conditioning operational variables for long runs.

Yoda et al. developed a continuous‐flow electrorotation device using comb‐shaped interdigitated electrodes on opposing substrates driven with 90‐degree phase‐shifted AC voltages (Figure 4c) [142]. The four‐electrode phasing on opposing substrates generates a rotating field that torques cells about a vertical rotation axis while electrokinetic alignment maintains positioning between electrode pairs (Figure 4c‐i). Electrode spacing strongly affects rotation magnitude because angular velocity scales with the square of electric field strength, and reducing the electrode gap was reported to increase rotation speed by approximately fourfold under otherwise comparable conditions [143]. Rotation rate was estimated from periodic similarity in the image stream using template matching, where the rotation period emerges as a repeating similarity pattern rather than explicit marker tracking (Figure 4c‐ii). This approach was validated by applying the same template‐matching logic to synthetically rotated images, yielding sub‐percent error and establishing that the analysis does not dominate measurement variance. Angular velocity was converted to the imaginary Clausius‐Mossotti factor after accounting for medium permittivity, viscosity, and field amplitude, and least‐squares fitting to a single‐shell model yielded membrane permittivity and cytoplasm conductivity (Figure 4c‐iii) [144]. Agreement between flow and no‐flow conditions supported the claim that continuous translation does not inherently bias recovered dielectric parameters when torque‐field geometry and medium properties are controlled. In array‐driven geometries, neighboring electrodes reversed the local rotation direction, and cells at alternating positions rotated clockwise and counterclockwise within the same field of view, providing an additional sanity check on field phasing and symmetry [142]. cROT establishes reproducible continuous‐flow dielectric characterization, but longitudinal monitoring of a biological process was not included in the reported implementation.

### 4.4 Dielectrophoretic Crossover Profiling

Dielectrophoretic profiling infers cell dielectric state by tracking how cells migrate in a spatially non‐uniform AC field, where the direction and magnitude of cell motion depend on the frequency‐dependent polarizability of the cell relative to the suspending medium [145, 146]. The physical basis is polarization of a cell relative to its suspending medium in an applied AC field, where a mismatch in complex permittivity creates an induced dipole and a spatial field gradient converts that dipole into a net force whose direction depends on the sign of the real Clausius‐Mossotti factor [147, 148]. The transition occurs at a crossover frequency where net dielectrophoretic motion changes sign [149]. In the point‐and‐planar microwell (PPM) implementation, crossover is determined from a frequency sweep spanning 1 kHz to 50 MHz, so the electrical phenotype is expressed as a single descriptor extracted from field‐driven motion in a defined medium. Under a single‐shell polarization model, crossover can be mapped to specific membrane capacitance when medium conductivity and permittivity are known, producing an interpretable proxy that can be compared with parameters obtained through other electrical modalities [150]. This crossover‐based descriptor can support label‐free single‐cell monitoring of longitudinal quality drift because the same motion assay can be repeated under standardized medium and field conditions to track distributional changes over time. Red blood cells (RBCs) are a relevant target because functional integrity changes during storage and handling, manifesting as gradual shifts in membrane and interior electrical behavior not readily tracked by staining‐based assays [151]. However, crossover is medium‐dependent, so small deviations in conductivity or temperature can masquerade as biological change unless calibration and logging are routine [152]. Stronger gradients improve sensitivity but can introduce electrode‐related artifacts or local heating. Crossover is also a compressed descriptor, so distinct physical changes can, in some cases, yield similar crossover shifts, which requires reporting uncertainty and retaining distribution‐level information for longitudinal comparison.

Oladokun et al. designed a PPM device where two perpendicular wire electrodes define a field geometry inside a variable‐height PDMS microwell, enabling adjustable inter‐electrode spacing without lithographic fabrication while preserving direct optical access to single‐cell trajectories (Figure 4d) [153, 154]. Each experimental run at one specific medium conductivity was completed in approximately one minute, supporting repeated measurements without long exposure that can bias crossover estimates. A non‐uniform field gradient near the point electrode and the expected directions of negative and positive DEP motion are summarized to define crossover as the frequency where net migration reverses (Figure 4d‐i). Directed migration and reversal were extracted from time‐lapse trajectories under stepped‐frequency forcing, enabling the sign change to be treated as a gating‐compatible motion phenotype. Erythrocytes were assayed under 4°C storage conditions across days 1 through 4. Erythrocyte accumulation near the point electrode in the microwell is directly observed during stepped‐frequency forcing, providing visual confirmation of field‐driven migration patterns (Figure 4d‐ii). A strong storage‐age dependence of the inferred membrane proxy was reported, with an approximately 3.8‐fold decline in specific membrane capacitance over four days under 4°C storage, consistent with progressive degradation of erythrocyte electrical integrity under pre‐analytical drift (Figure 4d‐iii) [155]. The same study demonstrated that osmotic state systematically shifts the apparent membrane proxy, with hypotonic swelling increasing and hypertonic crenation decreasing the inferred capacitance, and temperature profiling across a physiological‐to‐stress range further shifted the membrane proxy. The inferred specific membrane capacitance was compared with prior electrorotation benchmarks under matched medium assumptions. This comparison supports cross‐modality interpretability of the crossover‐derived membrane proxy within a defined calibration window [156]. This positions DEP crossover profiling as a pre‐analytical calibration and stress‐sensitivity framework, making explicit how storage age, osmotic state, and temperature can bias any dielectric inference if not standardized. DEP crossover profiling tracks dielectric changes across storage‐defined cohort time points, yet it does not constitute direct longitudinal monitoring of individual cells (Table 1).

**TABLE 1:** Summary of label‐free single‐cell monitoring platforms across imaging, vibrational spectroscopy, and electrical sensing‐based modalities.

Modalities	Platforms	Operational principles	State variables	Primary readouts	Acquisition formats	Throughputs/sampling intervals	Key performance metrics	Target cells	Refs.
Imaging	Holotomography	3D RI reconstruction	Structural (RI, morphology)	RI volume, CNN classification	Static, 3D time series	30 min intervals	99.3% accuracy^a^, 2–4 h lead^e^	HeLa, A549	[57]
	SLEC	Fabry‐Perot cavity lasing	Structural (nucleolus RI)	Lasing peaks, emission fingerprint	Static/Flow, 2D	2,000 eps	36x threshold contrast^c^, FWHM ∼0.07 nm^c^	K562, A549	[65]
	DLIC	Bright‐field nanojet lensing	Structural (size, eccentricity, RI)	Size, eccentricity, average RI	Flow, 2D snapshot	>270,000 cells	FDTD‐calibrated RI^c^, 3D state‐cloud	PBMCs	[73]
	NCC	SWCNT nIR fluorescence + lensing	Structural + chemical (H_2_O_2_ efflux)	Size, eccentricity, RI, H_2_O_2_ efflux	Flow, 2D time series	10^5^ cells/h	4‐phenotype panel, aging trajectory^e^	Human fibroblasts	[84]
Vibrational spectroscopy	FT‐CARS RFC	Coherent Raman (300–1600 cm^−1^)	Compositional (carbohydrates)	Raman spectrum, band integral	Flow, snapshot + sorting	∼50 eps	up to 98% purity^b^ (beads), ∼93% purity^b^ (cells)	Euglena, Microalgae	[96]
	pDEP‐DLD RFC	Spontaneous Raman (550–3100 cm^−1^)	Compositional (metabolic)	Full spectrum, learned embedding	Flow, spectral snapshot	30–2700 epm	>96% classification^a^, 5 h stable^d^	Yeast, algae, bacteria, cancer	[98]
	SRS	CH‐stretch SRS + phasor (2800–3050 cm^−1^)	Compositional (mitosis)	Phasor coordinates, band ratios	Static, 2D snapshot	Scan‐based (40‐image hyperspectral stack)	∼12.5% nuclear band change^e^, organelle mapping^c^	HeLa, SK‐BR‐3	[109]
	MIP‐QPI	MIR photothermal + QPI	Compositional (MIR absorption)	Phase amplitude, MCR components	Static, 2D time series	50 fps	Video‐rate imaging^d^, 40‐band MCR^c^	COS7	[115]
Electric	ISC	Broadband impedance (250 kHz‐550 MHz)	Dielectric (Csm, σcyto)	Double‐shell parameters	Flow, multi‐freq. sweep	250 cells/s	8‐freq simultaneous^c^, optimized fitting^c^	HL‐60, THP‐1	[129]
	piRT‐IFC	Dual‐freq impedance (100/180 kHz) + GPU	Dielectric (Csm, σcyto)	Real‐time intrinsic properties	Flow, time series	>100,000 cells/run	0.62 ms/cell^d^, KL<0.1 stability^d^	A549, 293T, HL‐60	[136]
	cROT	AC torque rotation (4‐phase)	Dielectric (εmem, σcyto)	Angular velocity, Im (CM)	Flow, rotation series	2,700 cells/h	32 parallel cells^c^, 4x speed gain	HeLa	[142]
	DEP	DEP crossover (1 kHz‐50 MHz)	Dielectric (Csm proxy)	Crossover frequency	Static, freq. sweep	∼1 min/run	3.8x Csm decline over 4 d storage^e^	Erythrocytes	[154]

^a^ Classification accuracy against supervised fluorescence or label‐based reference annotations.

^b^ Sorting purity defined by physical spectral gating criteria without label‐based ground truth.

^c^ Physical precision or resolution metric independent of biological classification.

^d^ Operational or temporal stability metric quantifying instrument consistency across acquisition cycles.

^e^ Biological sensitivity metric for state‐transition detection. Event, a single‐cell acquisition instance defined by platform‐specific triggering criteria including flow transit, DEP trapping, cavity interrogation, or constriction sealing. eps, events/s; epm, events/min; fps, frames/s; IFC, impedance flow cytometry.

## 5 Conclusions and Outlook

In conclusion, we reviewed representative platforms for multimodal label‐free single‐cell monitoring and organized them into three measurement families according to the intrinsic state variables. Imaging‐based platforms capture cell structural state variables through RI and morphology contrast, preserving spatial information from subcellular to population scales and enabling repeated observation without fluorescent labels [157]. Vibrational spectroscopy‐based platforms report cell compositional state variables through bond‐specific vibrational contrast, providing chemical fingerprints that encode the relative contributions of proteins, lipids, nucleic acids, and metabolites at single‐cell resolution. Electrical sensing‐based platforms recover cell dielectric state variables through frequency‐dependent AC‐field interactions, achieving high event rates without optical excitation while providing direct access to membrane capacitance and cytoplasmic conductivity. Table 1 summarizes the three modalities within a unified comparative framework, highlighting their underlying physical principles, representative state variables, performance characteristics, and major sources of drift relevant to longitudinal monitoring. Across the three modalities, the dominant physical and analytical constraints differ systematically. In imaging‐based platforms, reconstruction stability and illumination drift set the primary limits on repeated‐acquisition reproducibility. In vibrational spectroscopy‐based platforms, background fluorescence suppression and photon dose constraints govern the trade‐off between chemical specificity and acquisition speed. In electrical sensing‐based platforms, electrode interface drift and model identifiability in equivalent circuit inversion introduce the principal sources of parameter uncertainty. These modality‐specific constraints argue for integrative measurement architectures, in which rapid electrical or morphological readouts triage candidate states, and chemically specific or spatially resolved assays resolve ambiguities that no single modality can disentangle [158]. Across the representative platforms reviewed here, direct longitudinal monitoring is currently demonstrated only in a limited subset, whereas many others provide time‐ordered or monitoring‐relevant measurements without repeated single‐cell follow‐up.

For label‐free single‐cell monitoring to mature into a deployable analytical workflow, progress is required at multiple, interdependent levels spanning hardware, computation, and measurement governance. On‐site deployment requires hardware that is lighter, more compact, and robust enough to operate outside specialized laboratory environments while maintaining quantitative stability with minimal alignment and maintenance. This shift motivates simplified illumination and detection architectures, reliable sample handling under flow, and integrated computing pipelines that enable on‐device or edge inference under constrained network and power conditions. Molecular specificity remains a structural limitation because RI, impedance, and vibrational contrast each reflect overlapping contributions from multiple molecular components.

The use of machine learning in label‐free single‐cell monitoring is consequential for clinical and translational adoption because model outputs may either remain tied to physically interpretable features or arise from data‐driven representations without explicit biochemical attribution. Physically grounded models are independently verifiable against biophysical standards, whereas data‐driven classifiers can lose reliability under distributional shift in cell type, instrument state, or sample preparation. For deployment, interpretability, out‐of‐distribution robustness, and standardized reference datasets should therefore be treated as reporting requirements. Improving molecular attribution, therefore, increasingly relies on large reference datasets paired with orthogonal molecular assays to train artificial intelligence‐based, virtual staining‐like models that translate intrinsic signals into molecularly interpretable outputs. Calibration protocols and measurement reporting also need to be standardized so that uncertainty estimates, instrument state, and quality criteria accompany the extracted state variables together with recorded acquisition settings and medium conditions, enabling cross‐session and cross‐site comparability as platforms transition from research demonstrations to routine deployment.

Adequate validation of a label‐free monitoring platform requires three minimum components. First, orthogonal validation must confirm that observed feature shifts reflect biological state transitions rather than instrument‐level variation. Second, for longitudinal claims, temporal changes must be consistent with known biological kinetics and remain stable across independent acquisition sessions. Third, cross‐session reproducibility must be quantitatively established using defined metrics. Multimodal integration becomes most informative when sample handling is consistent, measurements are time‐aligned, and shared calibration references make structural, chemical, and dielectric readouts comparable, so that concordant shifts across modalities strengthen interpretation as true biological change while mismatched shifts flag modality‐specific instability for focused quality control. The practical relevance of label‐free single‐cell monitoring differs across application domains and maps directly onto the measurement capabilities reviewed here. At the same time, practical deployment remains constrained by modality‐specific trade‐offs in throughput, live‐cell compatibility, molecular specificity, and calibration robustness. High‐throughput flow‐compatible platforms are particularly relevant in oncology, where circulating tumor cell profiling and drug‐response heterogeneity analysis require repeated sampling of large cell populations without perturbing rare subpopulations. Immunological applications instead place greater value on sub‐minute temporal resolution and live‐cell compatibility, because transient activation states are poorly resolved by fixation‐based or endpoint assays. For cell manufacturing and therapy development, electrical and imaging platforms that support batch‐level distribution comparisons under standardized conditions are especially useful for quality assessment of stem cell and CAR‐T cell products, where reproducible population‐level readouts are prioritized over single‐cell trajectories.

When these capabilities are realized, calibrated label‐free trajectories can detect early state transitions in drug discovery, summarize clinically relevant heterogeneity in blood and tissue samples without labeling, and support routine process and release checks in cell manufacturing by tracking distribution shifts across batches without expanding marker panels. As label‐free platforms converge toward deployable, calibrated, and computationally efficient systems, they are positioned to redefine how cellular state is measured across the continuum from fundamental biology to clinical decision‐making, offering a path to quantitative single‐cell phenotyping that is scalable, non‐destructive, and independent of molecular labels.

## Conflicts of Interest

The authors declare no conflicts of interest.

## Data Availability

Data sharing not applicable to this article as no datasets were generated or analysed during the current study.
